# Knowledge and awareness of oral cancer: A cross-sectional survey in Trieste, Italy

**DOI:** 10.3389/froh.2023.1056900

**Published:** 2023-01-30

**Authors:** Katia Rupel, Matteo Biasotto, Margherita Gobbo, Augusto Poropat, Magdalena Theodora Bogdan Preda, Giuseppe Borruso, Lucio Torelli, Roberto Di Lenarda, Giulia Ottaviani

**Affiliations:** ^1^ Department of Medicine, Surgery and Health Sciences, University of Trieste, Trieste, Italy; ^2^ Unit of Oral and Maxillofacial Surgery, Ca’ Foncello Hospital, Treviso, Italy; ^3^ Department of Economics, Business, Mathematics and Statistics, University of Trieste, Trieste, Italy

**Keywords:** oral cancer, head and neck cancer, healthcare surveys, community surveys, behavior risk factors, preventive oral health

## Abstract

The aim of the study was to verify the knowledge on oral cancer and to assess possible differences in awareness and information basing on different demographic and subject-related factors. An anonymous survey was provided to 750 random subjects using online-based questionnaires. Statistical analysis was performed in order to evaluate the influence of demographic variables (gender, age, education) on knowledge of oral cancer and its risk factors. 68.4% of individuals knew about the existence of oral cancer, mostly from media and family/friends. Awareness was significantly influenced by gender and higher education, but not by age. Most participants recognized smoking as a risk factor, but alcohol abuse and sunlight exposure are less known, especially among less educated subjects. On the contrary, our study shows a diffusion of false information: more than 30% of the participants indicated the possible role of amalgam fillings in oral cancer onset, independently of gender, age or education. The results of our study suggest the need for oral cancer awareness campaigns, where school and healthcare professionals should be actively involved in promoting, organizing and finding methods to monitor the medium and long-term efficacy with proper methodological quality.

## Introduction

Oral squamous cell carcinoma (OSCC) represents one of the most common, yet scarcely known malignancies worldwide, affecting more than 300,000 individuals per year and causing 177,384 deaths annually, representing nearly 2% among all cancer sites (1). 9,700 new cases were reported in Italy in 2018, of them 7,400 males and 2,300 females (2). Recent epidemiological reports confirm that OSCC most commonly affects men over the fifth decade, with a rising trend in subjects younger than 45 years and in women worldwide (3). Despite general efforts to provide adequate interventions and therapies after diagnosis, OSCC mortality rates are remaining stable over time (about 50%), essentially depending on disease stage (4, 5). Alongside with exposure to risk factors and availability and access to clinical diagnosis and treatment, an important factor affecting mortality is the still scarce awareness of the pathology and its early signs, leading to important delays in diagnosis and worsening survival rates (6). Moreover, diagnosis in later stages also importantly affect the quality of life of the surviving patients (7) and increases the cost of care with prolonged hospitalization and the need for more complex surgical interventions and reconstructions (8). A recent systematic review investigated on the causes of delayed diagnosis in OSCC patients and concluded that the scarce knowledge of the population emerged as the main factor (9).

In accordance with other cancer types, the etiology of OSCC is strongly related to specific lifestyle habits and behavior, which include tobacco smoking, alcohol abuse, exposure to sunlight ultraviolet (UV) radiation, and human papilloma virus (HPV) infection (10). The latter is a particular entity, as it is mainly associated with oropharyngeal cancer (OPSCC) and affects younger patients than OSCC (11). Besides the knowledge about risk factors, which are common also to other cancer types, the scarce knowledge about the possibility of development of cancer inside the oral cavity remains a diffuse and major problem, alongside with the difficulty to identify a medical specialist to address in case of suspect symptoms (12).

Informative campaigns about OSCC and other cancer types characteristics and risk factors are performed worldwide, but their efficacy in reducing the time occurred by symptoms onset and referral to a proper physician are difficult to evaluate (13). The risk of such campaigns is missing specific groups of subjects that would particularly benefit from the information. Hence, assessment of OSCC knowledge among general population, verifying differential awareness in subcategories of people may be useful to identify specific population groups that need to be addressed with informative campaigns.

The aim of our study was to verify the knowledge about different aspects of OSCC and to assess possible differences in awareness and information basing on different demographic and subject-related factors such as age, gender and education level.

## Materials and methods

### Questionnaire development

A questionnaire on OSCC knowledge was designed by the clinical staff of the Oral Medicine and Pathology Unit (School of Dentistry, University of Trieste) and by the Scientific Promulgation Office (University of Trieste). Questions were developed basing on a previously employed questionnaire on OSCC knowledge among pre-adolescents and adolescents (14) and implemented including additional questions about the need for additional awareness campaigns. When asked about possible risk factors for OSCC, individuals had the possibility to indicate one or more among ascertained (cigarette smoking, alcohol consumption, sunlight exposure) or incorrect potential risk factors (amalgam fillings, fluoride-based products), in order to evaluate the diffusion of false information. The study met the ethical norms and standards stated in the Declaration of Helsinki and was approved by the local ethics committee (86/2018).

### Face and content validity of the questionnaire

The face validity of the questionnaire including appropriateness, logical sequence and comprehensibility of the questions was examined by 10 subjects (4 dentists, 4 dental hygienists and 2 students). The impact score (IS) of each item was calculated using a five-point Likert appropriateness scale ranging from 1 (not appropriate at all) to 5 (highly appropriate) and items scoring <1,5 were removed from the questionnaire (15). Subsequently, content validity was assessed using the content validity ratio (CVR) and content validity index (CVI). A panel of 10 experts in oral medicine rated each item as not necessary, useful but not essential, or essential and the CVR value was calculated following Lawshe's formula (16). Items scoring <0.62 were removed from the questionnaire. The same panel rated the relevance of each item in a four-point Likert scale and the CVI was calculated using the specific formula (17). Items scoring CVI < 0.80 were removed from the final questionnaire.

### Submission of the questionnaire

The questionnaire was distributed by students randomly to subjects with minimum age of 11 years visiting the University of Trieste stand during the “Trieste NEXT” 3-day long science dissemination event. Subjects were visitors of different ages randomly accessing a free-entrance pavilion set up in the context of a public dissemination initiative. Subjects were asked their consent to participate, and in case of acceptance were provided with a tablet to answer the items. A total of 750 questionnaires were completed. After the conclusion of the initiative, the data from the questionnaires were processed anonymously. Results were reported into a Microsoft Excel table and analyzed as frequency counts, percentages, and/or means.

### Statistical analysis

Statistical analysis was performed using R software, version 4.0.2 (R Foundation for Statistical Computing, Vienna, Austria. http://www.R-project.org/). Fishers’ exact test (for 2 × 2 contingency tables) was used to evaluate the association among different variables and OSCC awareness. All of the variables significantly associated with OSCC awareness were introduced into a multiple logistic regression model. Forward stepwise algorithms were used, with the rejection of those variables that did not fit the model significantly. Odds ratio (OR) and 95% confidence intervals (CIs) were also calculated. A *p*-value < 0.05 was used to reject the null hypothesis.

## Results

### Demographic characteristics of the participants

A total of 750 individuals completed the questionnaire. The demographic characteristics of the participants are reported in Table 1. The sample was further categorized into subgroups based on gender (male, female), age (<30 years, ≥30 years) and education level in order to verify which of these variables are associated to knowledge about OSCC and associated risk factors. To categorize patients according to education levels, the International Standard Classification of Education (ISCED) index has been employed. ISCED is the standard framework used to categorize and report cross-nationally comparable education statistics developed by the UNESCO Institute for Statistics (18). Specifically, ISCED levels 0–2 include primary and lower secondary education, while ISCED levels 3–8 include more than 8 years of education.

**Table 1:** Demographic characteristics of the participants and categorization in subgroups. Data are reported as frequency rates or mean ± standard deviation (for age).

Total number	750
**Gender (%)**	
Male	45.32%
Female	54.41%
**Age**	
Mean ± SD	32 ± 15
Range	10–92
**Age group (*n*, %)**	
<30 years	64.75%
≥30 years	35.25%
**Education (*n*, %)**	
ISCED 0–2	47.07%
ISCED 3–8	52.93%
**Has anyone among your family/friends experienced a cancer?**	
Yes	71.54%
No	28.46%

### Knowledge about OSCC, risk factors and sources of information

Results of the questionnaires are reported in Tables 2, 3. 68.4% of individuals knew about the existence of oral cancer, mostly from media (44.3%) and family/friends (34.5%), followed by school (21.0%) and their dentist (13.7%). While almost all participants were aware that smoking increases the risk for oral cancer (94.1%), nearly half (51.3%) had indicated alcohol consumption. Sunlight exposure was indicated by 15.4% of subjects, while a group of them wrongly indicated fluoride (12.7%) and amalgam fillings (34.7%). The majority of the subjects were aware that oral cancer is a malignant tumor (75.2%) and that a change in lifestyle habits can reduce the risk of developing the disease (83.6%). Subjects were generally unaware about survival rates, but 93.0% of them answered that early diagnosis can improve them.

**Table 2:** Knowledge about OSCC, possible risk factors, sources of information, possible interventions and their timing to contribute increasing awareness. Data are reported as frequency rates.

Question	Answer	
Which was the source of information about oral cancer?		
	Dentist	13.7%
	Family/Friends	34.5%
	School	21.0%
	Media	44.3%
	Other	11.8%
Do you think that changing lifestyle habits can reduce the risk of developing oral cancer?		
	Yes	83.6%
	No	13.6%
	Do not know	2.7%
Do you think that oral cancer has high survival rates?		
	Yes	20.8%
	No	22.4%
	Do not know	56.8%
Do you think oral cancer is a malignant tumor?		
	Yes	75.2%
	No	1.6%
	Do not know	23.2%
Do you think that an early diagnosis can improve survival rates?		
	Yes	93.0%
	No	2.0%
	Do not know	5.0%
Which professional figure would You turn on to with a suspect of oral cancer?		
	Dermatologist	4.6%
	General practitioner	36.1%
	Dentist	49.1%
	Oncologist	33.4%
	Otolaryngologist	35.4%
Do you think that there is need for additional knowledge/information about oral cancer?		
	Yes	98.2%
	No	1.8%
If yes, through which information channels?		
	Public meetings	36.0%
	Internet and social media	65.7%
	Newspapers and journals	31.6%
	School	76.5%
	Other	3.9%
What is the appropriate age to address the topic of cancer prevention? (years)		
	6–10	10.3%
	11–14	38.8%
	15–18	56.1%
	>18	22.9%

**Table 3:** Correlation of gender, age and education with knowledge about OSCC and its risk factors. Data are reported as frequency rates. A p-value < 0.05 was used to reject the null hypothesis. Significant results are highlighted in bold.

	Yes	No	*p*-value^a^	OR	CI	*p*-value^b^
**Have you ever heard about oral cancer?**						
Total	513 (68.4%)	237 (31.6%)				
Gender (*n*, %)						
Male	215 (29.3%)	119 (16.2%)	**0**.**02**	0.68	0.5,0.9	**0**.**02**
Female	291 (39.6%)	110 (15.0%)				
Age group (*n*, %)						
<30	324 (43.4%)	186 (21.3%)	0.32			
≥30	159 (24.9%)	77 (10.3%)				
Education (*n*, %)						
ISCED 0–2	224 (29.9%)	129 (17.2%)	**0.01**	1.54	1.1,2.1	**0.01**
ISCED 3–8	289 (38.5%)	108 (14.4%)				
**Do you think that smoking is a risk factor for oral cancer?**						
Total	697 (94.1%)	40 (5.9%)				
Gender (*n*, %)						
Male	306 (42.1%)	26 (3.6%)	**<0**.**05**	0.53	0.3,1	**<0**.**05**
Female	377 (51.9%)	17 (2.3%)				
Age group (*n*, %)						
<30	459 (62.3%)	23 (3.1%)	0.06			
≥30	234 (31.8%)	21 (2.8%)				
Education (*n*, %)						
ISCED 0–2	330 (44.5%)	21 (2.8%)	1			
ISCED 3–8	367 (49.5%)	23 (3.1%)				
**Do you think that alcohol is a risk factor for oral cancer?**						
Total	380 (51.4%)	357 (48.6%)				
Gender (*n*, %)						
Male	174 (24.0%)	158 (21.8%)	0.65			
Female	199 (27.4%)	195 (26.9%)				
Age group (*n*, %)						
<30	235 (31.9%)	247 (33.5%)	0.06			
≥30	143 (19.4%)	112 (15.2%)				
Education (*n*, %)						
ISCED 0–2	157 (21.2%)	194 (26.2%)	**<0**.**001**	1.65	1.2,2.2	**<0**.**001**
ISCED 3–8	223 (30.1%)	167 (22.5%)				
**Do you think that sunlight exposure is a risk factor for oral cancer?**						
Total	114 (15.4%)	623 (84.6%)				
Gender (*n*, %)						
Male	44 (6.1%)	288 (39.7%)	0.10			
Female	70 (9.6%)	324 (44.6%)				
Age group (*n*, %)						
<30	70 (9.5%)	412 (55.9%)	0.34			
≥30	44 (6.0%)	211 (28.6%)				
Education (*n*, %)						
ISCED 0–2	49 (6.6%)	302 (40.8%)	0.36			
ISCED 3–8	65 (8.8%)	325 (43.9%)				
**Do you think that fluoride is a risk factor for oral cancer?**						
Total	94 (12.7%)	643 (87.3%)				
Gender (*n*, %)						
Male	52 (7.2%)	280 (38.6%)	**0**.**02**	1.69	1.1,2.6	**0**.**02**
Female	39 (5.4%)	355 (48.9%)				
Age group (*n*, %)						
<30	72 (9.8%)	410 (55.6%)	**0**.**01**	0.51	0.3,0.8	**0**.**01**
≥30	21 (2.8%)	234 (31.8%)				
Education (*n*, %)						
ISCED 0–2	7.6%	39.8%	**0**.**02**	0.57	0.4,0.9	**0**.**01**
ISCED 3–8	5.1%	47.5%				
**Do you think that amalgam fillings are a risk factor for oral cancer?**						
Total	257 (34.7%)	480 (65.3%)				
Gender (*n*, %)						
Male	123 (16.9%)	209 (28.8%)	0.21			
Female	128 (17.6%)	266 (36.6%)				
Age group (*n*, %)						
<30	176 (23.9%)	306 (41.5%)	0.14			
≥30	79 (10.7%)	176 (23.9%)				
Education (*n*, %)						
ISCED 0–2	38 (16.7%)	295 (30.6%)	0.76			
ISCED 3–8	56 (17.9%)	352 (34.7%)				

^a^ For each variable, the Fisher's exact test was employed.

^b^ Significant results were further analyzed by means of a Multiple logistic regression analysis to obtain odds ratios (ORs) with 95% Confidence Intervals (CIs; lower – upper) and *p*-values.

When participants were asked which professional figure address in case of suspect of oral cancer, the most frequently chosen was the dentist (49.1%), followed by general practitioner, oncologist and otolaryngologist. Only 4.6% of the subjects would turn on to a dermatologist.

Almost all participants think that there is need for additional information about oral cancer (98.2%), mostly through school (76.5%), internet/social media (65.7%) and television (58.2%). In participants' opinion, the appropriate age to start addressing the topic of cancer and its prevention would be 15–18 years (56.1%), followed by 11–14 years (38.8%).

### Variables affecting knowledge about OSCC and its risk factors

Table 3 shows the results of the statistical analysis regarding the association of gender, age and education with knowledge about OSCC and its possible risk factors. Knowledge about the existence of oral cancer was significantly influenced by gender (Fisher's exact test *p* = 0.02) and education (Fisher's exact test *p* = 0.01), but not by age (Fisher's exact test *p* = NS). In particular, male subjects were less aware (Multiple logistic regression OR = 0.68; CI: 0.5;0.9 with *p* = 0.02), while participants with higher levels of education (Multiple logistic regression OR = 1.54; CI: 1.1;2.1 with *p* = 0.01) were significantly more informed.

Knowledge about cigarette smoking, as risk factor for OSCC development, was significantly affected by gender (Fisher's exact test *p* < 0.05), with male subjects being less aware (Multiple logistic regression OR = 0.53; CI: 0.3;1 with *p* < 0.05). Alcohol consumption was less frequently known in all subgroups, except for participants with higher education levels, whose knowledge was 1.65-fold higher than in participants with lower education levels (Fisher's exact test *p* < 0.001). The awareness of the correlation between sunlight exposure and OSCC was low in all subgroups without significant differences.

A proportion of participants wrongly indicated fluoride or amalgam fillings as risk factors for OSCC development. In particular, male subjects were more likely to indicate fluoride as a risk factor (Multiple logistic regression OR = 1.69; CI: 1.1;2.6 with *p* = 0.02), while older (Multiple logistic regression OR = 0.51; CI: 0.3;0.8 with *p* = 0.01) and highly educated (Multiple logistic regression OR = 0.57; CI: 0.4;0.9 with *p* = 0.01) participants were significantly better informed. However, there were not significant differences in knowledge about amalgam fillings not being associated with the risk of OSCC onset.

## Discussion

Large-scale awareness campaigns are extensively used to sensitize population on cancer prevention and screening for early diagnosis especially in the most frequent cancer types, leading to a decrease in their mortality (19). Early diagnosis is especially important in OSCC, as in first stages of the disease 80%–90% survival rates can be achieved, also minimizing the extent of surgery and fostering a better quality of life (20). However, global and national Italian cancer statistics are showing that both incidence and mortality are remaining stable over years (1, 2, 4, 5).

### Principal findings and comparison to other studies

One of the major issues is the generally scarce knowledge of the existence of oral cancer among both patients and clinicians, causing delays in the request for a specific professional assessment (21). The results of our survey confirm this trend, as only 68.4% of the 750 participants knew about the existence of oral cancer. Such knowledge rate is consistent with other data reported in literature among population studies on OSCC, where awareness rates were above 70% (22, 23) or around 50% (24, 25) respectively. However, the awareness rate in the included population is higher to the one we obtained from questionnaires submitted to preadolescent and adolescent participants to a campaign to increase awareness about oral cancer and its risk factors, performed in the same geographical area of North-eastern Italy (14). Specifically, the knowledge rate was of 26.8% among 460 students aged 12–14 years. We have previously hypothesized that the scarce knowledge about OSCC existence could be related to the age of participants, as previous studies included a population older than 18 years. The result of the present study seems to confirm this hypothesis, considering that we included subjects with older ages (32 ± 15).

When assessing which factors influenced the knowledge rate, consistently but surprisingly age did not turn to be a significant variable. Other variables significantly affected OSCC knowledge rate instead: male subjects were less likely to be informed, while higher education levels (ISCED 3–8) seem to foster knowledge and sensitization. This is an interesting result, as epidemiological data show that male gender has nearly double-fold incidence and mortality for OSCC with respect to female gender (1) and should be considered when preparing sensitization campaigns aimed at addressing the subjects at higher risk. There is evidence that men are generally less aware of cancer risk factors (26, 27) and tend to underutilize preventive health care services (28). Social disparities often play a major role in the accessibility to information and screening procedures. Other studies have investigated this topic with consistent results, as lower-income and lower-educated subjects were in general less likely to be screened for OSCC (29) and have a lower awareness (30, 31).

We observed a higher level of awareness about the importance of early diagnosis in improving survival rates, and about the importance of changing lifestyle habits in order to reduce the risk of developing oral cancer. Interestingly, there is a different level of awareness about risk factors. Subjects correctly identified smoking as the major risk factor (94.1%), consistently with the data obtained in our previous campaign among preadolescents (92.2%), and in a higher proportion compared with studies performed in Italy (32) and in other areas (22–24). Such data may suggest that previous campaigns performed on the territory were effective in informing and educating in identifying tobacco smoking as a risk factor for several cancer types, including OSCC in both younger and older subjects. When assessing the influence of demographic variables, male gender was associated with a lower knowledge of smoking being a risk factor for OSCC. In fact, male smokers are among the subgroups considered at high-risk (1, 2, 33), but apparently the less affected by previous campaigns and less likely to perform an oral cancer examination (34). Considering that smoking is inversely proportional to oral cancer awareness (35), it can be hypothesized that in our population a possible major prevalence of smokers among men may have contributed to our results.

Alcohol abuse is the other major risk factor for OSCC development, but it is generally scarcely recognized both in our (51.3%) and in other previously published studies (13, 17–19, 25, 32). Notably, higher education levels resulted associated to a major knowledge about alcohol abuse being associated to oral cancer, consistently with the study published by Hassona et al. (36). Few subjects (15.4%) identified sunlight exposure as a risk factor, without differences among population subgroups. Interestingly, other studies performed in Asian (31) and European (37) populations report similar frequencies.

Together with the scarce consciousness of ascertained risk factors, some media and uncontrolled information spreading often lead to consolidation of misbeliefs. The possible association between fluoride-based products and especially amalgam fillings with systemic diseases including malignancies, although not supported by scientific evidence, is unfortunately widespread. Our data confirm that both have been listed as potential risk factors for OSCC but to a different extent: while few subjects indicated fluoride (12.7%) without differences among subgroups, amalgam fillings were chosen by nearly one third of the participants (34.7%). Both proportions are higher than the ones obtained in our previous study conducted among preadolescents (14). In particular, the indication of amalgam wasn't influenced by any of the demographic variables, suggesting that this specific misbelief is uniformly distributed in general population. In contrast, older and highly educated female subjects were less likely to indicate fluoride.

The main source of information in our population were media, consistently with studies performed in other countries (30), followed by family/friends and school. Dentists are recognized as the main professional figures to turn on to in case of suspect of OSCC, although they provided information to only 13.8% of subjects; this gap should be bridged fostering the active participation of both hospital and private practice dental professionals in oral cancer awareness campaigns. On this point, our data differ from the results reported in a similar study performed on 600 subjects in the Naples region. Less than 30% of participants knew about the existence of oral cancer (consistently with our results), but among them 54.3% received specific information from their dentist (38). Almost all subjects included in our study recognize the need for additional information and encourage the dissemination especially through school *in primis*, followed by internet, social media and television. The appropriate age to start addressing cancer prevention issues, in our population's opinion, would be above 15 years. However, even preadolescents are already exposed to large amount of information about healthcare topics including cancer, possibly leading to misunderstandings and misbeliefs, being the reliability of the sources often difficult to understand. In our experience, such dissemination events and campaigns are fully understood and appreciated even in subjects aged 11–14 (14), encouraging the inclusion of younger subjects among target groups.

### Strengths and limitations

One of the major strengths of the study was the development of a questionnaire designed in order to evaluate knowledge and information of subjects starting from 11 years of age about oral cancer characteristics and risk factors. The questionnaire underwent face validity and content validity analysis and was filled completely by all subjects without the need for further explanations or help and is suitable to be employed also in other geographical areas following adequate translation. Results of the answers to the questionnaire provide an interesting picture confirming the overall trend of scarce knowledge about this specific topic, consistently with other studies.

Limitations of the study are the relatively small number of participants and the restricted geographical area (participants were all from Trieste, Italy or from other cities in the range of less than 50 km). Although interesting data have emerged (for example the age and education-related difference in knowledge, and the scarce information provided by dentists), further deepening and cause/effect relationships were not possible because specific questions were not included in the questionnaire.

### Implications and future directions

Social campaigns designed to raise awareness about the risk factors of different cancer types remain a widespread method to improve their prevention and/or promote early diagnosis (39). However, the proper understanding of media messages relating to cancer prevention and screening, alongside with the translation of knowledge into behavior changes are difficult to assess (40), especially considering the amount of unreliable information which leads to misunderstandings and misbeliefs. For example, a Cancer Research UK training workshop performed to increase awareness of cancer screening programs and risk factors, confirmed its success at a 2-month follow-up (13), but there is limited data about medium and long-term impact, especially regarding oral cancer. Moreover, a recent overview confirms how social campaigns usually increase knowledge of the disease and attendance at health services in the short-term, often obtaining a response from subjects at lesser risk (41). Direct reminders, small media, and provider audit and feedback appeared to be effective interventions to increase the uptake of screening for three cancers (breast, cervical, colorectal) (42).

Taking into account the geographical and numerical limitations, the results of our study suggest how there is definitely the need for oral cancer awareness campaigns, where school and healthcare professionals (in this case dentists), appropriately trained and informed, should be actively involved in promoting, organizing and finding methods to monitor the medium and long-term efficacy of cancer prevention dissemination campaigns, with proper methodological quality. The possible interventions may include direct contact, mass media, small media or group education initiatives and further studies are needed to compare their effectiveness.

## Data Availability

The raw data supporting the conclusions of this article will be made available by the authors, without undue reservation.
